# Evaluation of a novel anti-oxidant Ubiquinol acetate (EnQ10) for its safety by *in vitro* methods

**DOI:** 10.1016/j.heliyon.2024.e39826

**Published:** 2024-10-24

**Authors:** Mohan Cheluru Umesh, K.M. Geetha, Srinivas Seekallu

**Affiliations:** a Department of Pharmacology, College of Pharmaceutical Sciences, Dayananda Sagar University, Bengaluru-560068, Karnataka, India; b Anthem Biosciences Pvt. Ltd., Bommasandra Industrial Area, Phase 1, Bommasandra, Bengaluru-560099, Karnataka, India

**Keywords:** Coenzyme Q10, Ubiquinol acetate, Reconstructed human epidermis, Reconstructed human corneal-like epithelium, Phototoxicity, Mouse lymphoma assay

## Abstract

**Background:**

As an active form of Coenzyme Q10 (CoQ10), Ubiquinol acetate (EnQ10) is a newly developed, chemically stable, and physiologically active novel antioxidant. In addition to its anti-oxidant properties, CoQ10 is a useful cosmetic agent for human skin.

**Purpose:**

This study goal was to examine the safety of EnQ10 using *in vitro* models instead of animal models. The models that were used for cosmetic and genotoxicity testing were approved by the OECD.

**Methods:**

The study included testing for a number of toxicological endpoints, including skin irritation test (OECD TG 439), skin corrosion test (OECD TG 431), eye irritation test (OECD TG 492), phototoxicity test (OECD TG 432), and Mouse lymphoma assay for *in vitro* cell gene mutation test (OECD TG 490). Regulatory authorized EpiSkin^TM^-SM skin model (for skin irritation/corrosion); SkinEthic^TM^-HCE model (for eye irritation); BALB 3T3 NRU phototoxicity assay (for phototoxicity test); and mouse lymphoma assay (for TK gene mutation test) are among the series of chosen *in vitro* models that were conducted as per GLP standards.

**Results:**

In terms of cosmetic toxicity and genotoxicity, none of the tests that were run produced positive results for EnQ10.

**Conclusion:**

As an alternatives to animal model procedures, we utilized extensive, well known *in vitro* methodologies to analyze the health effects of EnQ10, a new antioxidant used in cosmetic and nutraceutical products. These *in vitro* investigations showed that ubiquinol acetate (EnQ10) is safe for genotoxicity, phototoxicity, skin and ocular irritation/corrosion.

## Highlights

• Ubiquinone, the inactive oxidized form, and Ubiquinol, the active reduced form, are the two forms of coenzyme Q10 that are readily available.
• Ubiquinol (active reduced form) exhibits chemical instability.
• Recently, ubiquinol acetate (EnQ10) was discovered as a stable new antioxidant.
• Coenzyme Q10 and its derivatives' antioxidant qualities have been shown to have numerous positive impacts on human skin.
• On the other hand, no prior research has been done on the cosmetic safety or toxicological characteristics.
• Alternative *in vitro* test methods are more dependable and widely recognized models for evaluating the safety of cosmetics.

## 1 Introduction

Coenzyme Q10 (CoQ10) is an endogenous antioxidant found in nearly all human tissues and is involved in the mitochondria of cells energy production. CoQ10 is found chemically in two forms: ubiquinol, which is an active reduced form, and ubiquinone, which is an inert oxidized form. Humans possess antioxidant qualities due to ubiquinol's several modes of action. Despite being more advantageous as an antioxidant, it is chemically unstable and quickly oxidizes when exposed to air, changing into the inactive form of ubiquinone (Matsuo et al. 2016). In order to overcome the chemical stability and increase biological activity, ubiquinol acetate (EnQ10), a salt version of ubiquinol, was developed recently [1,2].

For over four decades, coenzyme Q10, also known as ubiquinol or ubiquinone, has been a popular recommendation for long-term exogenous oral nutritional supplementation in humans. Coenzyme Q10 and its derivatives are employed as cosmetic agents in addition to being used as dietary supplements. Numerous studies have shown that applying CoQ10 directly to the skin can minimize cellular damage or hormonal imbalances by promoting antioxidant protection and cumulative energy production in skin cells [3]. EnQ10 has undergone evaluation in genotoxicity and animal safety tests recently; however, no safety information is known for its cosmetic application. Therefore, using the 3Rs approach, the current study assessed for the mutagenicity and cosmetic safety of EnQ10 for use in cosmetics using a battery of alternative *in vitro* methods chosen in accordance with OECD test guidelines ((Skin irritation test-OECD TG 439), (Skin corrosion test-OECD TG 431), (Eye irritation test-OECD TG 492), (Phototoxicity test-OECD TG 432), and (Mouse lymphoma assay-OECD TG 490). All the studies were performed in accordance with GLP standards.

## 2 Materials and Methods

### 2.1 Test Compound

The test compound, ubiquinol acetate (EnQ10), was a gifted sample (Batch No.- A31600427; Purity by HPLC-98.2 %) from Anthem Biosciences Pvt. Ltd.'s Chemistry department in Bengaluru, India.

### 2.2 Media and Reagents

4-Nitroquinoline-N-Oxide (catalog no. N8141; Sigma Aldrich), Aroclor 1254 Induced male Sprague Dawley rat liver S9 (catalog no. 11–101; Moltox), Benzo[a]pyrene (catalog no. B1760; Sigma Aldrich), Chlorpromazine hydrochloride (catalog no. C8138; Sigma Aldrich), DMEM-high glucose (catalog no. D7777; Sigma Aldrich), DPBS (catalog no. D8537; Sigma Aldrich), EBSS (catalog no. E2888; Sigma Aldrich), Fischer's medium (catalog no. AT054; HiMedia), Glacial acetic acid (catalog no. 1930020521; Merck), Horse Serum (catalog no. 16050; GIBCO), Hypoxanthine (catalog no. H9636; Sigma Aldrich), Isopropyl alcohol (catalog no. P0731; Rankem), Methotrexate (catalog no. M9929; Sigma Aldrich), Methyl acetate (catalog no. 809711; Merck), MTT (catalog no. M2128; Sigma Aldrich), MycoAlertTM Mycoplasma Detection Kit (catalog no. LT07-318; Lonza), Neutral red solution (catalog no. N2889; Sigma Aldrich), Penicillin-Streptomycin 100X solution (catalog no. 15140; GIBCO), Pluronic F-68 solution 10 % (catalog no. TCL023; HiMedia), RPMI-1640 medium (catalog no. R6504; Sigma Aldrich); Sodium bicarbonate (catalog no. S6014; Sigma Aldrich), Sodium dodecyl sulfate (catalog no. L3771; Sigma Aldrich); Sodium pyruvate solution (catalog no. S8636; Sigma Aldrich), Thymidine (catalog no. T1895; Sigma Aldrich), Trifluorothymidine (catalog no. T2255; Sigma Aldrich).

### 2.3 *In Vitro* Tests for Irritation/Corrosion

*Skin irritation/corrosion*: The supplier of EpiSkin^TM^-SM kits (RhEs) was Episkin (Lyon, France). RhE tissues (Batch no: 18-EKIN-028 and 18-EKIN-031) were received in well-accepted condition. After being removed from the agar media, the EpiSkin tissues were placed on 12-well tissue culture plates and a maintenance medium was added. following an overnight preincubation period at 37 °C with 5 % CO_2_. For test compound EnQ10, preliminary MTT reduction and coloring potential studies were carried out. The irritation and corrosion tests were conducted within the expiry date of the EpiSkin™-SM kits.

*Skin irritation test:* Test compound EnQ10 (10 mg per tissue), negative control (DPBS, 10 μL per tissue), and positive control (5 % w/v SDS, 10 μL per tissue) were applied in triplicate on an epidermal tissue surface and incubated for 15 min at room temperature (RT) in accordance with OECD TG 439 [4] for the irritant test. Tissue inserts were exposed, cleaned with DPBS, and then post-incubated in an incubator set at 37 °C with 5 % CO_2_ for 42 h. Following the post-incubation phase, the percentage of viable cells in the treated epidermal inserts was ascertained using the MTT assay.

*Skin corrosion test:* Test compound EnQ10 (20 mg per tissue, n = 2 per incubation time point), negative control (9 g/L NaCl solution, 20 μL per tissue, n = 2), and positive control (Glacial acetic acid, 20 μL per tissue, n = 2) were applied on the surface of epidermal tissue and incubated for 3, 60, and 240 min at RT in accordance with OECD TG 431 for the corrosion test [5]. Tissue inserts were exposed, rinsed with DPBS, and the MTT assay was run to find the percentage of viable cells in the treated epidermal inserts.

*Eye irritation test*: The SkinEthic^TM^- HCE kit (RhCEs) was procured from Episkin in Lyon, France. RhCE tissues (Batch no.: 18-HCE-039) were received in well-accepted condition. After being taken out of the agar medium, SkinEthic-HCE tissues were placed in 6-well tissue culture plates and maintenance medium was added. following an overnight preincubation period at 37 °C with 5 % CO_2_. For the test chemical EnQ10, preliminary MTT reduction and coloring potential tests were carried out. The eye irritation test was conducted within the expiry date of SkinEthic™- HCE kit.

The test substance EnQ10 (30 mg per tissue), the negative control (DPBS, 30 μL per tissue), and the positive control (methyl acetate, 30 μL per tissue) were applied in duplicate on the epithelium tissue surface in accordance with OECD TG 492 [6] for the eye irritation test. The samples were then incubated for 4 h at 37 °C with 5 % CO_2_. Following exposure, tissue inserts underwent DPBS rinsing, 30 min of medium post-soaking, and 18 h of incubator post-incubation (37 °C, 5 % CO_2_). Following the post-incubation phase, the percentage of viable cells in the treated epithelial inserts was ascertained using the MTT assay.

#### 2.3.1 MTT Assay for Irritation/Corrosion Tests

EpiSkin tissue inserts were inserted into 12-well tissue culture plates together with 2 mL of 0.3 mg MTT/mL MTT solution for skin irritation/corrosion testing. The plates were then incubated for 3 h at 37 °C and 5 % CO_2_. For tissues, a biopsy punch was used, and the entire surface of the epidermis was extracted using acidified isopropanol (0.04 N HCl) to remove formazan formation that resulted from the vitality of the cells.

In order to test for eye irritation, treated SkinEthic-HCE tissues were put into each well of a 6-well tissue culture plate, which contained 1 mL of a 1 mg/mL MTT solution. The plates were then incubated for 3 h at 37 °C and 5 % CO_2_. Using isopropanol, the whole surface of the epithelium was stripped of the formazan that resulted from the vitality of the cells.

After adding the prepared formazan extract to a 96-well culture plate, the absorbance was measured with a VarioSkan microplate reader (Thermo Fischer Scientific, Finland) at 570 nm. The percentage of cell viability in relation to the negative control, as specified by the OECD 431, 439, and 492 TGs, was ascertained using the measured absorbance values.

### 2.4 Test Conditions for Phototoxicity Test

In preliminary test, the spectral analysis of Ubiquinol acetate was analyzed in different pH conditions as per OECD TG 101 [7].

BALB/c 3T3, clone A31 cell line (catalog no. CCL-163^TM^) was procured from ATCC. In a humidified environment of 5–7.5 % CO_2_ at 37 °C, BALB/c 3T3 cells were cultured in Dulbecco's modified eagle's medium (DMEM) supplemented with 10 % (v/v) heat-inactivated Newborn calf serum (NBCS), 2 mM L-glutamine, 0.17 M sodium bicarbonate, penicillin (100 IU/mL), and streptomycin (100 μg/mL). The cell line was examined to make sure mycoplasma infection wasn't present. For the phototoxicity experiment, the suggested passage of less than 100, which is not sensitive to UVA radiation at 5 J/cm^2^ (% cell viability more than 80 %), was utilized.

In accordance with OECD TG 432 [8] for the phototoxicity experiment, BALB/c 3T3 cells were trypsinized after reaching 60–70 % confluency. Trypan-blue was used to verify the viability of the cells, and the cells were seeded at a density of 1 X 10^4^ cells/well into 96-well culture plates (2 plates per compound) and incubated for an entire night. Following a wash with Earl's Balanced Salt Solution (EBSS, 150 μL/well), the cells were exposed to eight different concentrations of EnQ10 (62.5, 31.25, 15.625, 7.813, 3.906, 1.953, 0.977, and 0.488 μg/mL with 1 % v/v Ethanol); in addition, 100 μL/well of chlorpromazine hydrochloride (CPZ) (Positive control at 100, 30, 10, 3, 1, 0.3, 0.1, and 0.03 μg/mL with 1 % v/v DMSO) was added, and the plates were incubated for 1 h using a CO_2_ environment.

The experiment also comprised blank wells and vehicle controls (untreated) groups (1 % v/v ethanol for EnQ10; 1 % v/v DMSO for CPZ). Then, using a solar simulator (SOL-500, Honle UV, USA), one plate per compound was exposed to UVA (UVA+) for 50 min at an intensity of 1.7 mW/cm^2^, and a second plate was utilized for dark condition (UVA−) by retaining the plate at room temperature for 60 min. Afterwards, the plates underwent EBSS washing, DMEM culture media (100 μL/well) was added, and the CO_2_ incubator was allowed to incubate for 20 h.

#### 2.4.1 NRU Assay for Phototoxicity Test

After the cells were rinsed with EBSS, neutral red (50 μg/mL) solution was added to each well, and the plate was incubated for 3 h in the CO_2_ incubator. Under the inverted microscope, the cells were examined.

After carefully washing the cells with EBSS, the neutral red supernatant solution was decanted. Each well received 150 μL of neutral red desorb solution (50 ethanol: 1 glacial acetic acid: 49 water), which was then mixed with a thermomixer (Eppendorf) and agitated for 10 min at 300 rpm. Using a VarioSkan microplate reader, the absorbance of the plate was measured at 540 nm. The software Phototox Version 2.0 was utilized to compute the photo irritation factor (PIF) and mean photo effect (MPE) based on the acquired absorbance values.

### 2.5 Mouse Lymphoma Assay (*In Vitro* Cell Gene Mutation Test)

The L5178Y TK ± clone (3.7.2C) cell line was procured from ATCC (catalog no. CRL-9518TM). Fischer's medium was used to cultivate and suspend the L5178Y TK ± clone (3.7.2C) murine lymphoma cells [9]. Additional ingredients were L-glutamine (2 mM), penicillin (100 U/mL), streptomycin (100 μg/mL), sodium pyruvate (1 mM), and Pluronic-F68 at 0.1 % v/v. Every day, cultures were diluted to 2 × 10^5^ cells/mL in order to avoid overgrowth (>10^6^ cells/mL). The L5178Y TK ± clone (3.7.2C) cell line was examined for mycoplasma contamination, model chromosomal number (n = 40), and doubling time. To reduce the background mutant frequency (MF), the characterized cells were cleansed to remove any TK−/−mutants that may have existed beforehand. First, cells were treated for one day at 3, 5, 0.1, and 7.5 μg/mL with THMG (Thymidine-Hypoxanthine-Methotrexate-Glycine) and subsequently with THG (Thymidine-Hypoxanthine-Glycine). For cleansing, cells were treated with THMG (Thymidine-Hypoxanthine-Methotrexate-Glycine at 3, 5, 0.1, and 7.5 μg/mL respectively) for 1 day and then THG (Thymidine-Hypoxanthine-Glycine at 3, 5 and 7.5 μg/mL respectively) for 2 days.

After a preliminary solubility test, the lowest insoluble concentration of 62.500 μg/mL (1 % v/v Acetone) was found to be soluble in ubiquinol acetate, which was shown to be visibly soluble at 31.250 μg/mL. Six test concentrations of EnQ10 at 62.500 to 1.953 μg/mL (1 % v/v Acetone) were used in the dose range-finding study on L5178Y TK ± clone (3.7.2C) cells at 6 X 10^6^ cells/culture, in the absence (∼4h and ∼24h treatment) and in the presence (∼4h treatment) of metabolic activation system (S9). For the metabolic activation system (S9 mix, 10 % v/v), the NADPH regeneration system (NRS) was constructed in accordance with Maron & Ames' instructions (Mutat. Res. 113:173, 1983) [10]. After assessing each culture's suspension growth (SG1 and SG2), cells were plated.

Based on the results from the dose range-finding study, four doses selected for the mutagenicity test (main study) evaluation were 62.500, 31.250, 15.625, and 7.813 μg/mL in duplicate culture (N = 2). In the mutagenicity study, the L5178Y TK ^±^ clone (3.7.2C) cells at 6 X 10^6^ cells/culture were treated with Ubiquinol acetate concentrations as mentioned above for ∼4 h in the absence of metabolic activation system and ∼4 h in presence of metabolic activation system. 4-Nitroquinoline-N-Oxide at 0.1 μg/mL (4 & 24 h in the absence of S9) and Benzo[a]pyrene at 2.5 μg/mL (4 h in presence) were used as positive controls for the experiment.

## 3 Results

### 3.1 Irritation/Corrosion Tests

According to preliminary testing (coloring potential and MTT reduction tests), EnQ10 was neither a coloring potential agent nor an MTT reducer. The findings of skin irritation/corrosion tests using RhE tissues and ocular irritation tests using RhCEs tissues, as indicated in Table 1, indicate that EnQ10 does not irritate or corrode skin or eye tissues. EnQ10's percentage cell viability is comparable to that of the negative control.

**Table 1:** % Cell Viability of *In Vitro* Skin/Eye Irritation and Corrosion Tests.

	**Group ID**	**Skin Irritation Test**			
		Mean OD ± SD	Mean %cell viability ± SD	Classification	Cut-off value
	Negative control (DPBS)	0.650 ± 0.03	100.00 ± 4.19	Non-irritant	Non-irritant/No category: Mean tissue cell viability should be >50 %
	Positive control (5 % SDS)	0.068 ± 0.06	10.41 ± 8.99	Irritant	
	EnQ10	0.648 ± 0.05	99.74 ± 7.13	Non-irritant	
	**Group ID**	**Skin Corrosion Test**			
		**Mean OD ± SD**	**Mean %cell viability ± SD**	**Classification**	**Cut-off value**
	Negative control (9 g/L NaCl)	1.040 ± 0.022	100.00 ± 2.109	Non-corrosive	Non-corrosive/No category: Mean tissue cell viability should be ≥ 35 % after 240 min exposure
	Positive control (Glacial Acetic acid)	0.028 ± 0.007	2.69 ± 0.631	Corrosive	
	EnQ10 - 3 min	1.019 ± 0.034	97.98 ± 2.752	Non-corrosive	
	EnQ10 - 60 min	1.010 ± 0.025	97.12 ± 2.448	Non-corrosive	
	EnQ10 - 240 min	1.071 ± 0.064	102.98 ± 6.119	Non-corrosive	
	**Group ID**	**Eye Irritation Test**			
		**Mean OD**	**Mean %cell viability**	**Classification**	**Cut-off value**
	Negative control (DPBS)	2.208	100.00	Non-irritant	Non-irritant/No category: Mean tissue cell viability should be >50 %
	Positive control (Methyl acetate)	0.026	1.15	Irritant	
	EnQ10	2.076	94.02	Non-irritant	

### 3.2 Phototoxicity Test

**Fig. 1:**
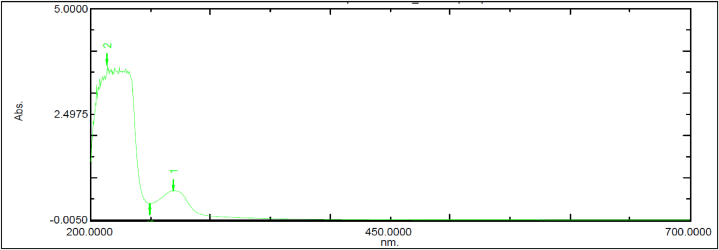
Spectral Analysis of Ubiquinol Acetate (EnQ10) in Neutral pH.

**Fig. 2:**
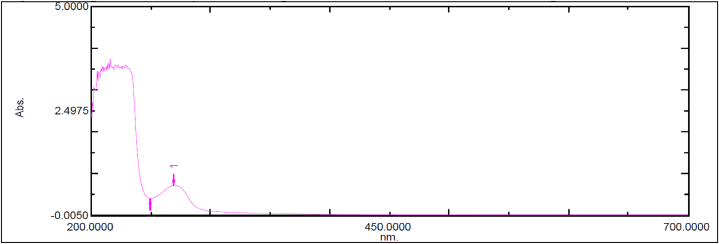
Spectral Analysis of Ubiquinol Acetate (EnQ10) in Acidic pH.

**Fig. 3:**
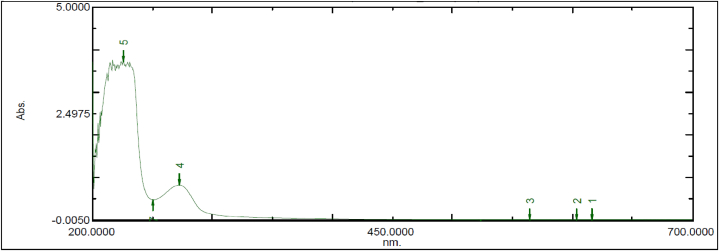
Spectral Analysis of Ubiquinol Acetate (EnQ10) in Neutral pH.

EnQ10's spectra investigation showed that it is a UV-active molecule (Fig. 1, Fig. 2, Fig. 3). Its molar absorption coefficient was also determined to be greater than 1000 L mol^−1^ cm^−1^. Therefore, the molecule was considered to assess its potential for phototoxicity.

In primary phototoxicity test, EnQ10 was determined to be non-cytotoxic at all tested concentrations, and it was impossible to determine the IC_50_ values in both the presence and absence of UVA radiation. Because of this, the Photo Irradiation Factor (PIF) was calculated to be PIF = 1 (Table 2), and the mean photo effect (MPE) was discovered to be −0.034.

**Table 2:** Photo Irritation Factor (PIF) and Mean Photo Effect (MPE) Values.

Group ID	3T3 NRU Phototoxicity		
	PIF	MPE	Prediction
Chlorpromazine HCl (CPZ)	25.315	0.201	Phototoxic
EnQ10	PIF = 1 (Calculated PIF)	−0.034	Non-Phototoxic

### 3.3 Mouse lymphoma assay (*In vitro* cell gene mutation test)

In the dose range-finding experiment, EnQ10 was determined to be non-cytotoxic at all tested concentrations, according to relative total growth (RTG). In the primary investigation, no tested ubiquinol acetate concentration showed an absolute increase in TK mutants when S9 was present (4 and 24 h) or not (Table 3). Up to 62.5 μg/mL of EnQ10, no statistically significant changes in the frequency of mutants were seen at any tested dose levels. Every value in the treated groups was comparable to the values in the negative control group.

**Table 3:** Mouse Lymphoma Assay - Relative Total Growth and Mutation Frequency.

A. Dose Range Finding Study			
Group ID	Relative Total Growth (RTG)		
	4 h (-S9)	4 h (+S9)	24 h (-S9)
Vehicle control (1 % Acetone)	100.00	100.00	100.00
Media control (Fischer Medium)	106.35	121.76	106.70
Ubiquinol acetate			
1.953 μg/mL	111.33	109.14	95.00
3.906 μg/mL	72.45	110.84	101.04
7.813 μg/mL	76.21	112.65	90.77
15.625 μg/mL	76.48	91.33	96.06
31.250 μg/mL	80.95	91.41	73.52
62.500 μg/mL	67.66	81.90	78.77

**B. Mutagenicity Test (Main Study)**								
**4 h Exposure (-S9)**			**24 h Exposure (-S9)**			**4 h Exposure (+S9)**		
**Group ID**	**%RTG**	**MF**	**Group ID**	**%RTG**	**MF**	**Group ID**	**%RTG**	**MF**
Vehicle control (1 % Acetone)	128.12	56.3	Vehicle control (1 % Acetone)	92.23	118.1	Vehicle control (1 % Acetone)	79.53	105.2
	73.14	87.4		108.02	91.2		119.39	60.6
Media control (Fischer Medium)	119.29	60.2	Media control (Fischer Medium)	114.97	113.5	Media control (Fischer Medium)	95.86	70.5
	92.21	69.7		118.8	90.1		82.30	77.6
Ubiquinol acetate			Ubiquinol acetate			Ubiquinol acetate		
7.813 μg/mL	77.37	82.5	7.813 μg/mL	81.25	78.8	7.813 μg/mL	78.17	78.1
	81.19	106.6		67.24	65.8		84.03	84.2
15.625 μg/mL	90.62	94.5	15.625 μg/mL	73.02	74.8	15.625 μg/mL	80.68	82.0
	67.89	103.4		62.93	107.5		68.19	68.4
31.250 μg/mL	78.63	82.4	31.250 μg/mL	83.39	78.6	31.250 μg/mL	64.61	74.5
	77.64	110.0		74.48	94.6		56.37	108.1
62.500 μg/mL	97.00	55.2	62.500 μg/mL	36.04	139.6	62.500 μg/mL	76.81	74.9
	81.15	63.1		68.36	113.6		74.50	92.7
4-Nitroquinolene			4-Nitroquinolene			Benzo[a]pyrene		
0.1 μg/mL	30.06	1255.1^a^	0.1 μg/mL	11.91	2620.8^a^	2.5 μg/mL	9.21	2411.1^a^
	32.34	1376.4^a^		9.06	2287.4^a^		13.06	1766.6^a^

Note.

a indicates p-value was found to be statistically significant compared to vehicle control.

## 4 Discussion

This work offers comprehensive, widely-accepted methods for evaluating the potential health concerns of a novel antioxidant called EnQ10, which is widely used as a cosmetic and nutraceutical product, *in vitro* as an alternative to animal model procedures. In order to provide more accurate responses, we have attempted to highlight the scientific research gaps in the literature results on CoQ10 derivatives in this study. Numerous antioxidant compounds are helpful to the skin and are commonly found in cosmetic items intended for human use. Every cosmetic product that is produced and sold must be safe for use on human health, according to regulations governing cosmetics. Numerous cosmetic creams intended for topical application containing Coenzyme Q10 [11] or its derivatives are available in the market. Despite the positive impact these products had on the skin, there is evidence that topically applied hydroxydecyl ubiquinone can cause allergic contact dermatitis in people, making their safe use questionable [12]. In another instance, when ubiquinone-10 (uq-10, Coenzyme Q10) was assessed for phototoxicity, it revealed increased amounts of superoxide and singlet oxygen [13]. Additionally, it was difficult to manufacture stable CoQ10-formulated cosmetics in the past.

It has been demonstrated that using CoQ10 as a dietary supplement improves blood pressure, oxidative stress, diabetes, infertility, and heart disease in people [[14], [15]]. Therefore, it's critical to demonstrate ubiquinol's safety for long-term use in humans.

According to the current experiment's *in vitro* alternatives' results from skin/eye irritation, skin corrosion, phototoxicity, and genotoxicity, ubiquinol acetate is safe for human skin and eyes and has no negative impacts on irritation, corrosion, phototoxicity. Furthermore, ubiquinol acetate was found to be non-mutagenic at the TK locus at the studied dose levels, according to data from the mouse lymphoma experiment. Comparably, in genotoxicity tests such as the *in vitro* micronucleus test, the *in vitro* chromosomal aberration test in the CHO-K1 cell line, and the bacterial reverse mutation test in *Salmonella typhimurium* strains, non-genotoxic outcomes were noted for ubiquinol acetate by Gajanan Deshmukh et al [1].

## 5 Conclusion

This work offers comprehensive, widely recognized methods for evaluating the potential health effects of ubiquinol acetate, a new antioxidant that is widely utilized in cosmetic and nutraceutical products, *in vitro* as an alternative to animal model procedures. We have attempted to provide more accurate responses by concentrating on scientific gaps in the literature on CoQ10 derivatives. The results of a battery of *in vitro* experiments confirmed the safety of ubiquinol acetate (EnQ10) with respect to genotoxicity, phototoxicity, skin and ocular irritation/corrosion.

## CRediT authorship contribution statement

**Mohan Cheluru Umesh:** Writing – original draft, Formal analysis, Data curation. **K.M. Geetha:** Writing – review & editing, Supervision, Conceptualization. **Srinivas Seekallu:** Validation, Project administration, Investigation.

## Data availability statement

Data will be made available on request.

## Funding

During the preparation of this publication, the authors state that no grants, funding or other forms of support were received.

## Declaration of competing interest

The authors declare that they have no known competing financial interests or personal relationships that could have appeared to influence the work reported in this paper.
